# Adapting the AD8 and CDR for Samoan and Tongan‐Speaking Populations: A Validation Study

**DOI:** 10.1002/alz70857_107641

**Published:** 2025-12-26

**Authors:** Justina P Tavana, John Kauwe, JoAnn T. Tschanz

**Affiliations:** ^1^ Brigham Young University, Provo, UT, USA; ^2^ Utah State University, Logan, UT, USA

## Abstract

**Background:**

In the realm of cognitive health assessment and dementia research, the recognition of cultural and linguistic diversity among populations has emerged as an imperative consideration. Alzheimer's disease and related dementias (ADRD) pose a global health challenge, affecting individuals from diverse backgrounds. To ensure equitable and accurate assessments of cognitive impairment, it is essential to develop and validate assessment tools that are adapted for the communities they serve. Pacific Islanders, both in their homelands and diaspora, have distinct cultural and linguistic characteristics that require tailored tools. Among the largest Pacific Islander groups in the U.S., Samoans and Tongans also have the highest number of native speakers, highlighting the need for culturally appropriate cognitive assessments.

**Methods:**

We recruited 26 participants (15 Samoan, 11 Tongan), aged 50 or older and fluent in Samoan or Tongan, from diasporic communities in Utah and Hawaii. Recruitment strategies included brain health events, social media, local organizations, churches, news outlets, and word‐of‐mouth referrals. Trained bilingual interviewers conducted data collection and administered cognitive assessments.

**Results:**

The study validated the Samoan and Tongan versions of the AD8 and CDR through rigorous translation, back‐translation, and expert review. Focus group feedback ensured cultural relevance and clarity. Interrater reliability indicated perfect agreement for the AD8 and strong agreement for the CDR in both languages. Both instruments exhibited robust psychometric properties, with significant correlations among the Samoan assessments (AD8‐SMN and CDR‐SMN: r=0.70, *p* = 0.004; AD8‐SMN and MoCA: r=0.78, *p* = 0.0008; CDR‐SMN and MoCA: r=‐0.53, *p* = 0.042). Strong correlations were also observed among the Tongan assessments (AD8‐TNG and CDR‐TNG: r=0.751, *p* = 0.008; AD8‐TNG and MoCA: r=‐0.83, *p* = 0.002). However, the CDR‐TNG and MoCA did not correlate significantly (*r* = ‐0.39, *p* = 0.232), likely due to the small sample size.

**Conclusions:**

This study addresses a critical gap in dementia research among minority populations. The validated Samoan and Tongan versions of the AD8 and CDR demonstrate strong psychometric properties, providing reliable tools for early detection, accurate diagnosis, and effective ADRD management. These tools provide a valuable resource for improving cognitive health outcomes and ensuring more equitable dementia care.

